# Distinct Clinical and Biological Features of Diffusely Metastatic Versus Bulky Localized Lung Cancer: Real-World Outcomes from a University Cancer Center in Germany

**DOI:** 10.3390/cancers17233728

**Published:** 2025-11-21

**Authors:** Blerina Resuli, Julia Walter, Diego Kauffmann-Guerrero, Jürgen Behr, Paola Arnold, Jeremias Götschke, Gabriela Leuschner, Julia Kovács, Chukwuka Eze, Christian Schneider, Amanda Tufman

**Affiliations:** 1 Department of Medicine V, University Hospital, LMU Munich, Ziemssenstraße 1, 80336 München, Germany; julie.walter@gmx.de (J.W.);; 2 Comprehensive Pneumology Center Munich (CPC-M), German Center for Lung Research (DZL), Bavarian Cancer Research Center (BZKF), 81377 Munich, Germany; 3 Department of Thoracic Surgery Munich, University Hospital, LMU Munich, 80336 Munich, Germany; 4 Department of Radiation Oncology, University Hospital, LMU Munich, 80336 Munich, Germany

**Keywords:** lung cancer, tumor size, bulky, diffusely metastatic, CRP, LDH

## Abstract

**Background:** Lung cancer metastasis results from local invasion, loss of adhesion, migration, and distant implantation. Some small tumors metastasize diffusely other tumors progress to local bulky disease without metastasis. Here we compare these biologically divergent courses of progression and present an analysis of clinical features related to early diffuse metastasis vs. bulky disease. **Methods**: We retrospectively collected data from pathologically confirmed not-metastatic “bulky” (cT3-T4cN0cM0) or “diffusely metastatic” (cT1-T2cN1-3cM1) patients with lung cancer treated at our center from 2013 to 2023. Clinical data including sex, age, histology, ECOG-PS, tumor stage, smoking status, presence, and site of metastases, LDH, CRP, NLR ratio were collected. **Results**: A total of 375 patents with “bulky” (*n* = 95) or diffusely metastatic lung cancer (*n* = 280) were included in the analysis. In the univariate analysis, the diffusely metastatic population was younger (*p* = 0.001), had a higher proportion of never smokers (*p* = 0.02), histologically adenocarcinoma (*p* < 0.0001), TTF1 positive (*p* < 0.00001) and elevated baseline LDH in serum (LDH > 250U/l, *p* < 0.0001) compared to “bulky” population. Patients with diffuse metastatic disease presented worse OS (HR = 2.34, *p* = 0.0001) and PFS (HR = 6.89, *p* < 0.0001) compared to patients with bulky disease. **Conclusions**: Younger age, never smokers, TTF1 positivity, poorly differentiated tumors, adenocarcinoma histology were independently associated with risk of distant metastasis.

## Simple Summary

Lung cancer can follow different patterns of progression. Some tumors spread early and diffusely to distant organs, while others grow locally into bulky masses without metastasizing. In this study, we compared these two distinct clinical courses and identified characteristics associated with early metastatic spread, including younger age, certain histological features, and molecular markers. These patients also experienced worse overall and progression-free survival compared to those with bulky tumors. Understanding these differences can help guide treatment decisions and improve personalized care for patients with lung cancer.

## 1. Introduction

The immense global burden of cancer continues to claim millions of lives each year, representing one of the most significant public health challenges of our time [1,2]. Among these, lung cancer stands out due to its aggressive biology, high mortality, and frequent late-stage diagnosis, leading to profound social and economic consequences [3,4]. Despite advances in multimodal therapy, many patients still experience early metastatic dissemination, underscoring the need to understand the biological factors that determine disease progression and prognosis [5,6,7,8].

The biological mechanisms underlying lung cancer metastasis are complex, involving sequential processes such as local invasion, epithelial-to-mesenchymal transition (EMT), intravasation, survival in circulation, and colonization of distant organs [9,10]. Clinically, metastatic behavior is heterogeneous: some small primary tumors disseminate widely at an early stage, whereas certain large “bulky” tumors remain locally confined [11,12]. Tumor size remains a key prognostic factor and central to the TNM staging system; however, the relationship between tumor size, molecular characteristics, and risk of early metastasis is not fully understood [13,14].

This gap in understanding highlights the necessity of a detailed comparison between biologically distinct NSCLC phenotypes, specifically locally advanced bulky tumors and small tumors with early diffuse metastases. Such knowledge could inform prognostic stratification and personalized therapeutic strategies, particularly in settings where comprehensive molecular profiling is not readily available [15,16,17].

In this retrospective study, we aimed to fill this gap by systematically examining two distinct NSCLC progression phenotypes:

1. Locally advanced, bulky, non-metastatic tumors (cT3–T4, cN0, cM0);
2. Small primary tumors with early diffuse metastases (cT1–T2, cN1–3, cM1).

We evaluated a comprehensive set of clinical and biological endpoints, including patient demographics, tumor histology, molecular and genomic profiles, laboratory biomarkers (such as LDH, CRP, and NLR), and patterns of disease progression. By comparing these phenotypes, we sought to identify clinical and molecular factors associated with local confinement versus early systemic dissemination, thereby providing novel insights into lung cancer biology and potential prognostic indicators.

## 2. Materials and Methods

### 2.1. Study Design and Study Population

This retrospective study was conducted at Ludwig-Maximilians-Universität (LMU) Hospital, Munich, using medical records from patients treated between January 2013 and December 2022. Two cohorts were analyzed: (1) patients with non-metastatic “bulky” cT3–cT4cN0cM0 disease, and (2) patients with small primary tumors but diffusely metastatic cT1–cT2cN1–3cM1 disease, according to the AJCC 8th edition.

The definition of “bulky” versus “diffusely metastatic” disease was chosen to contrast two distinct biological phenotypes: patients with cT3–cT4cN0cM0 tumors typically present with large, locally advanced primary lesions without distant spread, reflecting predominant local aggressiveness, whereas cT1–cT2cN1–3cM1 cases are characterized by small primary tumors but systemic dissemination, representing an early and diffusely metastatic phenotype. This dichotomous classification aimed to isolate these two biologically different behaviors. Intermediate presentations, such as locally advanced but node-positive or oligometastatic disease, were intentionally excluded to avoid biological overlap and ensure clearer comparison between the two patterns of tumor progression.

Inclusion criteria were: age > 18 years, histologically confirmed lung cancer, and at least one antitumor treatment (chemotherapy, immunotherapy, radiotherapy, or surgery). Exclusion criteria included: age < 18 years, absence of therapy, other AJCC stages, cancer of unknown primary, lung metastases from other tumors, or concurrent bulky (cT3–T4) and metastatic (cM1) disease. This ensured comparison between biologically distinct phenotypes—localized bulky tumors versus small metastatic tumors.

Clinical data including sex, age, tumor histology, tumor stage, performance status measured in Eastern Cooperative Oncology Group (ECOG) score, Lactate Dehydrogenase (LDH) levels, C—reactive protein (CRP) serum levels, neutrophils-to-lymphocyte (NLR) ratio and common driver mutations such as epidermal growth factor receptor (EGFR) and anaplastic large cell lymphoma (ALK). Laboratory parameters were collected at various time points during the course of treatment, rather than strictly prior to therapy initiation or within two weeks of pathological diagnosis, to reflect a broader clinical context.

Given the retrospective design, treatment regimens were heterogeneous and not consistently documented across the cohort and thus were not used as a variable in the analysis. Although treatment information was available for most patients, the type, sequence, and duration of therapies (chemotherapy, radiotherapy, immunotherapy, or surgery) varied considerably over the study period and between institutions. Therefore, inclusion of treatment as a covariate would have introduced significant confounding and bias. Survival analyses focused on baseline clinical and biological characteristics rather than treatment-specific effects.

The study was conducted in accordance with the Declaration of Helsinki and approved by the Institutional Ethics Committee of the University Hospital of Munich (LMU), (Approval-code: 476-16 UE, approval date 5 August 2016).

### 2.2. Statistical Analysis

Patient and tumor characteristics are presented as absolute and relative frequencies and as median with standard deviation and compared using Student’s *t*-test and Chi-squared test, respectively. We also performed checks for normality and variance homogeneity before applying parametric tests.

We used logistic regression analysis to identify factors associated with having T1–T2 metastatic disease compared to T3–T4 tumors without lymph node or distant metastases. Variables were selected based on clinical relevance and significance in univariate analysis. Multicollinearity among covariates was assessed to ensure model stability.

Kaplan–Meier curves display differences in univariate overall survival (OS) and progression-free survival (PFS) depending on tumor size. Differences in survival curves were tested using the Log-Rank test. The proportional hazards assumption for Cox regression was assessed using Schoenfeld residuals.

Multivariate Cox regression analysis was used to analyze differences in OS and PFS according to tumor size, adjusted for confounding variables including age, sex, performance status, histology, and selected laboratory biomarkers. Missing data were handled by complete-case analysis, and sensitivity analyses were performed to confirm the robustness of results.

A significance threshold of alpha < 0.05 was applied for all analyses. All analyses were performed using R Studio (R version 4.0), and tables and figures were created in Microsoft Excel and R Studio.

## 3. Results

### 3.1. Patient Characteristics

Overall, 375 patients with “bulky” (*n* = 95) or diffusely metastatic (*n* = 280) lung cancer were included in our analysis (Table 1). Patients were predominantly male (57.9% in the diffusely metastatic cohort and 63.2% in the bulky cohort) and ever smokers (68.6% in the diffusely metastatic cohort and 81% in the bulky cohort). Median age was 64 years (range 53–75) in the diffusely metastatic cohort, and 68.4 years (range 58.7–78.1) in the bulky cohort.

Of them, 151 patients (53.9%) with diffusely metastatic disease had ECOG-PS 0 and 52 patients (54.7%) in the “bulky” population (Table 1).

Of 95 patients with “bulky” disease, 34.7% had adenocarcinoma histology, 47.4% had squamous non-small cell lung cancer, and 2.1% had small-cell lung cancer (SCLC). Among 280 patients with diffusely metastatic disease, 70.4% presented with adenocarcinoma histology, 8.9% with squamous NSCLC, and 10.4% with SCLC (Table 2).

Among patients with stage diffusely metastatic disease, therapy was considered appropriate in 177 cases (63.2%) and inappropriate in 102 cases (36.4%), based on a combination of clinical and molecular criteria (Table 3). In the subgroup of patients with bulky disease, therapy was identified as appropriate in 62 cases (65.3%) and inappropriate in 33 cases (34.7%) (Table 3). Therapy appropriateness was determined using predefined criteria that incorporated compliance with established clinical guidelines for stage and histology, integration of molecular profiling results (e.g., NGS-based detection of actionable mutations) when available, and multidisciplinary expert consensus in cases where guidelines or molecular data did not provide a definitive recommendation. Although statistical analysis revealed no significant differences in therapy appropriateness between the two groups (*p* = 0.84), it is important to note that approximately one-third of patients in both groups received therapy considered inappropriate. This relatively high proportion may confound survival analyses, as outcomes could be influenced by treatment quality rather than intrinsic tumor biology. These findings suggest that the proportion of patients receiving appropriate therapy, whether assessed through conventional clinical evaluation or integrating NGS-based molecular assessment, is comparable across stages and disease burden categories.

Detailed patient characteristics and therapy appropriateness stratified by histology (NSCLC vs. SCLC) are presented in the Supplementary Material (Tables S1–S3). Specifically, Table 3 shows that therapy appropriateness differed markedly between histological subtypes: 59.8% of NSCLC patients with T1–T2 tumors and 64.5% with T3–T4 tumors received appropriate therapy, whereas among SCLC patients this proportion reached over 90% in both groups. Although these differences did not reach statistical significance (*p* = 0.52), they indicate a trend toward higher treatment adequacy in SCLC compared with NSCLC, a trend that should be interpreted with caution given the overall variability in therapy appropriateness.

### 3.2. Univariate Analysis of Association Between Tumor Characteristics and Tumor Size

In the univariate analysis, patients with diffusely metastatic disease were significantly younger than those with bulky tumors (mean age 64.0 vs. 68.4 years, *p* = 0.001, Table 1). A higher proportion of metastatic patients were never-smokers (16.4% vs. 4.2%, *p* = 0.02), while smoking history was more frequent in the bulky cohort. Histologically, adenocarcinoma predominated in the metastatic cohort (70.4% vs. 34.7%, *p* < 0.0001), whereas squamous-cell carcinoma was more common in bulky tumors (47.4% vs. 8.9%, *p* < 0.0001, Table 2).

Biological markers also revealed distinct associations. TTF1 expression was markedly enriched in diffusely metastatic tumors (57.8% vs. 20.0%, *p* < 0.00001, Table 2). Poorly differentiated histology (grade 3) was more frequent in metastatic disease (51.4% vs. 34.7%, *p* = 0.01). LDH levels at diagnosis were significantly higher in metastatic patients (mean 291.9 vs. 170.9 U/L, *p* < 0.0001), with 47.8% of metastatic cases showing LDH ≥ 250 U/L, compared to only 17.9% in bulky disease. By contrast, CRP and NLR values did not differ significantly between groups.

Regarding molecular alterations, EGFR mutations (13.2% vs. 2.1%, *p* < 0.0001) and ALK rearrangements (5.4% vs. 0%, *p* < 0.0001) were strongly associated with diffusely metastatic tumors, while TP53 co-mutations were more frequently in the bulky cohort (7.4% vs. 3.2%, *p* = 0.14) (Table 2).

Detailed tumor characteristics stratified by histology (NSCLC vs. SCLC) are presented in the Supplementary Material (Table S2).

### 3.3. Multivariable Analysis of Association Between Tumor Characteristics and Tumor Size

In multivariable logistic regression analyses (Table 4), several independent predictors of diffuse metastatic presentation were identified. Increasing age was inversely associated with the likelihood of presenting with small primary tumors and widespread metastases (odds ratio [OR] = 0.97 per year, *p* = 0.04), suggesting that younger patients are more prone to this aggressive disease phenotype. Similarly, smoking status emerged as a protective factor: both former smokers (OR = 0.19, *p* = 0.03) and current smokers (OR = 0.20, *p* = 0.03) demonstrated significantly lower odds of diffuse metastatic presentation compared with never-smokers.

Histological subtype exerted a substantial influence on metastatic risk. Patients with squamous-cell carcinoma were markedly less likely to present with small, diffusely metastatic tumors relative to those with adenocarcinoma (OR = 0.11, *p* < 0.0001), underscoring the heterogeneity of metastatic potential across lung cancer histologies. Among laboratory parameters, elevated LDH was the most potent predictor of the diffuse metastatic phenotype. Specifically, patients with LDH ≥ 250 U/L exhibited an 8.5-fold higher likelihood of presenting with small primary tumors and distant metastases (*p* < 0.0001), whereas those with intermediate LDH levels (100–249 U/L) had a 2.8-fold increased risk (*p* = 0.01), compared with patients with LDH < 100 U/L.

Conversely, higher levels of C-reactive protein (CRP) were inversely associated with diffuse metastatic spread (OR = 0.97 per mg/L, *p* = 0.02), suggesting that systemic inflammation may contribute preferentially to bulky local tumor growth rather than early dissemination. Collectively, these findings indicate that diffuse metastatic presentation is influenced by a combination of patient-specific, histological, and biochemical factors, highlighting distinct biological and clinical pathways underlying tumor dissemination.

### 3.4. Univariate and Multivariate Analysis of Survival

**Figure 1:**
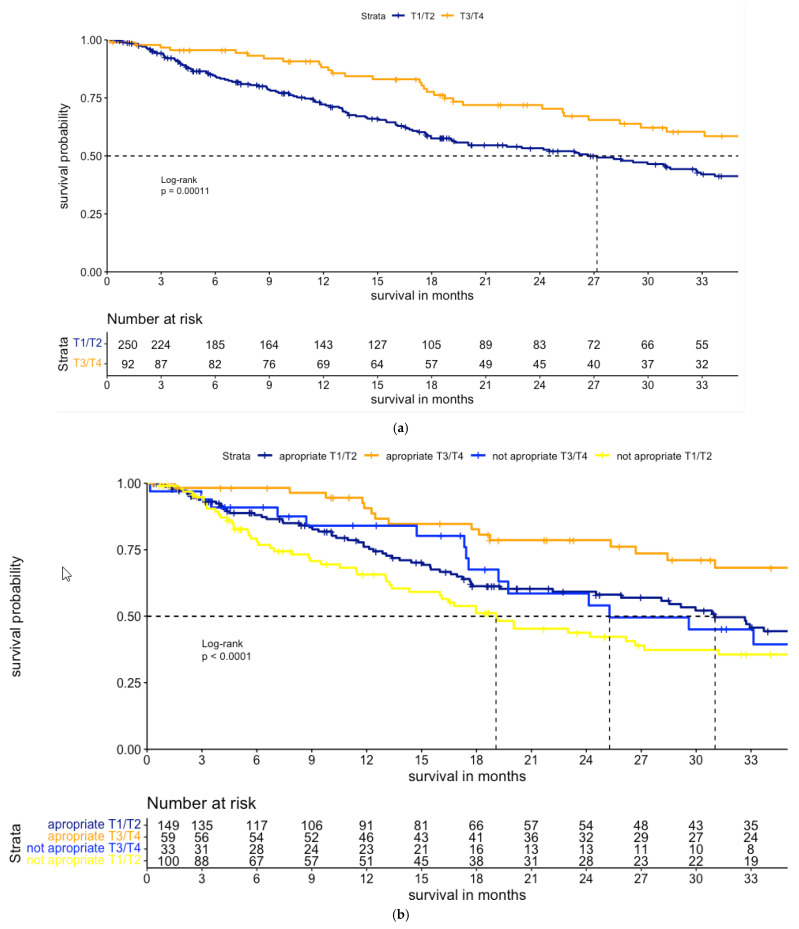

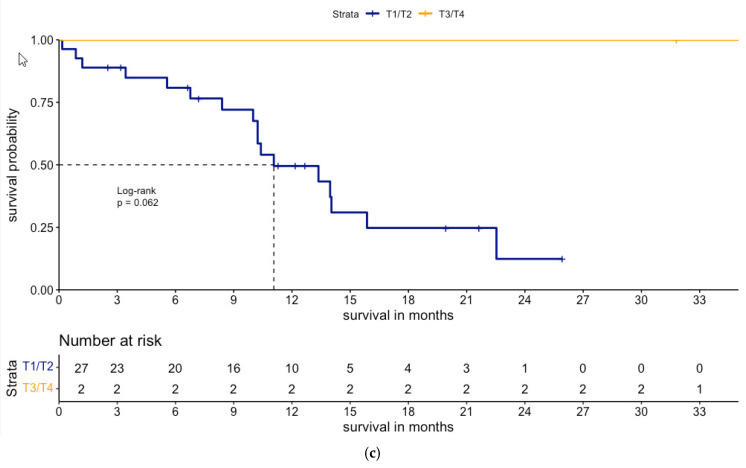
(**a**) Kaplan–Meier curves of OS. (**b**) Kaplan–Meier curves of OS based on treatment appropriateness. (**c**) Kaplan–Meier Analysis of Overall Survival in SCLC.

**Figure 2:**
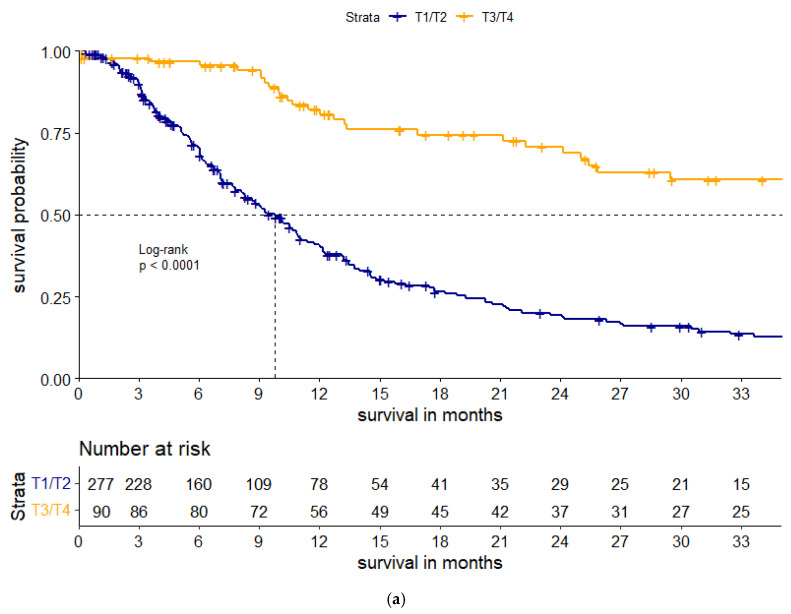

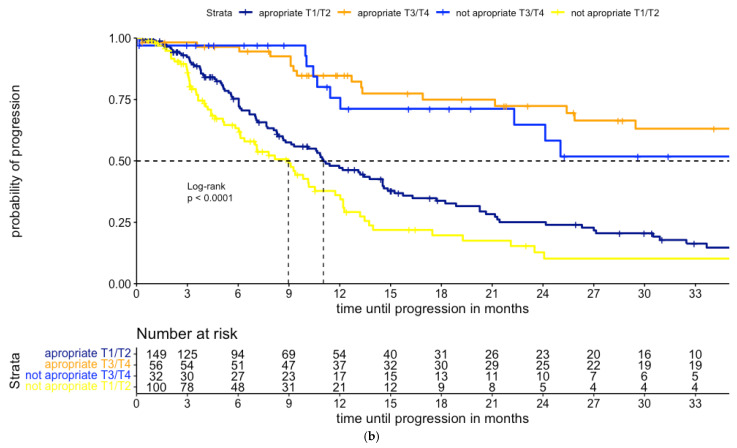
(**a**) Kaplan–Meier Analysis of Progression-Free Survival. (**b**) Kaplan–Meier curves of PFS based on treatment appropriateness.

Kaplan–Meier survival analyses demonstrated pronounced differences in outcomes between the two defined clinical phenotypes (Figure 1 and Figure 2). Patients presenting with bulky tumors experienced significantly improved overall survival (OS) compared with those exhibiting small, diffusely metastatic disease, with median OS of 22.3 months versus 8.7 months, respectively (log-rank *p* < 0.0001). Similarly, progression-free survival (PFS) was markedly prolonged in the bulky tumor cohort, with a median of 14.1 months compared with 3.2 months in patients with small, disseminated disease (log-rank *p* < 0.0001). Notably, these survival advantages persisted in subgroup analyses stratified according to the appropriateness of therapeutic intervention (Figure 1b and Figure 2b), as well as within the SCLC subgroup (Figure 1c), underscoring the robustness of these findings across clinically relevant subpopulations.

Kaplan–Meier analyses stratified by histology (NSCLC vs. SCLC) further demonstrated the prognostic divergence between the two tumor phenotypes (Supplementary Figures S1 and S2).

Among NSCLC patients, bulky tumors were associated with significantly improved OS and PFS compared with diffusely metastatic disease (median OS 22.8 vs. 8.5 months, *p* < 0.0001). In contrast, within the SCLC subgroup, survival outcomes were uniformly poor, but patients with localized bulky disease still exhibited numerically longer OS (10.4 vs. 6.1 months, *p* = 0.07).

When stratifying by therapy appropriateness, patients receiving appropriate treatment—according to molecular or clinical criteria—consistently experienced superior outcomes (Figure 1b and Figure 2b), irrespective of histological subtype. These findings emphasize that both tumor phenotype and adequacy of systemic therapy are independent determinants of survival across lung cancer histologies.

Multivariable Cox proportional hazards regression analyses further confirmed that tumor phenotype constitutes an independent predictor of survival, even after adjustment for established prognostic covariates (Table 5 and Table 6). Specifically, patients with small primary tumors accompanied by diffuse metastases exhibited an increased risk of mortality compared with individuals harboring bulky tumors (hazard ratio [HR] = 2.22, 95% confidence interval [CI] 1.42–3.46, *p* = 0.0004). Moreover, this phenotype was associated with a strikingly elevated risk of disease progression, with a nearly sevenfold higher likelihood of progression relative to bulky tumor counterparts (HR = 6.58, 95% CI 3.96–10.94, *p* < 0.0001).

In addition to tumor phenotype, several other clinical parameters were independently associated with adverse OS. Male sex (HR = 1.51, *p* = 0.01), poor performance status as defined by ECOG PS ≥ 2 (HR = 3.90, *p* < 0.0001), and elevated serum lactate dehydrogenase (LDH ≥ 250 U/L; HR = 1.65, *p* = 0.03) were all significantly predictive of inferior survival outcomes. Additional negative prognostic factors included smoking history (former smokers: HR = 2.14, *p* = 0.01; current smokers: HR = 2.25, *p* = 0.03). Interestingly, patients with intermediate LDH levels (100–250 U/L) demonstrated a protective effect on PFS (HR = 0.51, *p* = 0.002), potentially identifying a biologically distinct subgroup with more favorable disease kinetics. Former smokers (HR = 1.67, *p* = 0.03) and current smokers (HR = 1.61, *p* = 0.04) were negative prognostic factors for PFS. Additionally elevated CRP (HR = 1.01, *p* = 0.01) negatively impacted PFS.

## 4. Discussion

Lung cancer remains a leading cause of cancer-related mortality worldwide and continues to pose a substantial global health burden [11,12]. Over the past decade, multimodal treatment strategies—including surgery, radiotherapy, chemotherapy, targeted therapy, and immunotherapy—have improved survival for selected patient populations [13,14]. Despite these advances, durable clinical benefit is often confined to patients with specific molecular or immunological characteristics [15], and predictive biomarkers in routine practice remain suboptimal [16,17]. In this context, clinical and pathological variables may serve as complementary tools for patient stratification, particularly where comprehensive molecular profiling is not readily accessible [18,19,20].

In this retrospective analysis, we examined prognostic determinants associated with two distinct disease progression patterns: bulky locally advanced tumors without distant metastases versus small primary tumors presenting with early diffuse metastases. While tumor size is a recognized prognostic factor in both NSCLC and SCLC [21,22,23,24,25,26], the paradox of large tumors remaining locally confined while smaller primaries disseminate extensively remains poorly understood. Enhancing our understanding of these divergent phenotypes may inform biologically driven treatment strategies, minimize unnecessary toxicity, and refine personalized care.

Our findings indicate that bulky tumors were associated with older age, smoking history, squamous-cell histology, TTF1 negativity, and elevated CRP, whereas diffusely metastatic disease was more common in younger patients, adenocarcinoma histology, TTF1 positivity, poorly differentiated tumors, elevated LDH, and oncogenic driver mutations (EGFR, ALK).

The role of TTF1 merits particular attention. Historically considered a favorable prognostic marker, emerging evidence suggests TTF1 may facilitate metastatic dissemination through lymphatic invasion and distant organ involvement [27,28,29]. TTF1 regulates transcriptional programs influencing epithelial–mesenchymal transition (EMT), cellular adhesion, and surfactant protein production, promoting plasticity and motility. Activation of the PI3K/AKT and Wnt/β-catenin pathways in TTF1-positive tumors may further enhance invasion and metastasis [30,31]. These findings indicate that while TTF1 positivity may retain prognostic value in early adenocarcinoma, its biological role is context-dependent and may contribute to systemic dissemination in advanced disease.

Similarly, elevated LDH, reflecting tumor burden and glycolytic activity, has been consistently linked to poor outcomes [32,33]. High LDH supports proliferation and survival via lactate accumulation, extracellular matrix degradation, and immune evasion [34,35], and was strongly predictive of a diffusely metastatic phenotype in our cohort.

Conversely, bulky tumors were associated with chronic inflammation, as indicated by elevated CRP and smoking history. CRP reflects systemic inflammation, nutritional status, and immune competence. Chronic inflammation contributes to local tumor growth through sustained cytokine signaling (IL-6, TNF-α), fibroblast activation, and angiogenesis, but does not necessarily drive metastasis [36,37,38]. These observations suggest that bulky tumor growth may be primarily driven by tumor–microenvironment interactions, whereas early systemic dissemination is more closely linked to intrinsic tumor biology and molecular alterations [39,40,41,42,43].

It is important to emphasize that this study establishes associative, rather than causal, relationships. Residual confounding is an inherent limitation of retrospective analyses, and unmeasured variables—such as microenvironmental composition, immune status, and comprehensive molecular signatures—may influence observed patterns.

This study clarifies prognostic differences between bulky localized tumors and small primaries with early diffuse metastases, linking clinical, pathological, and molecular features to distinct disease trajectories. Bulky tumors were associated with local inflammatory factors, while early metastatic disease correlated with TTF1 positivity, elevated LDH, and oncogenic driver mutations, highlighting divergent underlying biology. These findings generate biologically plausible hypotheses for tumor progression and provide practical clinical tools, as TTF1, LDH, and CRP are easily measurable biomarkers that can support patient stratification, guide surveillance, and inform treatment decisions. Prospective validation may enable phenotype-specific, biomarker-driven management in lung cancer.

Several limitations merit consideration. First, the exclusion of patients with bulky primaries and synchronous metastases limits generalizability and precludes analysis of this “intermediate” group. Second, treatment heterogeneity inherent to retrospective real-world cohorts may have influenced survival outcomes. Third, the relatively low utilization of NGS, particularly in earlier study periods, likely limited detection of actionable mutations and underestimated the prognostic impact of molecular subtypes. Additionally, due to heterogeneous and incompletely documented treatment protocols, reliable subgroup analyses assessing therapeutic impact were not feasible.

We acknowledge that our findings are associative and do not imply causality. Future prospective studies with more balanced cohorts are warranted to validate these observations and further investigate the biological determinants of local tumor aggressiveness versus early systemic dissemination. Integrating multi-omics approaches in such studies may also clarify the role of biomarkers such as TTF1, LDH, and CRP in guiding phenotype-specific, biomarker-driven management strategies.

## 5. Conclusions

Our analyses demonstrate that clinical features such as younger age, never-smoking status, TTF1 positivity, and adenocarcinoma histology are strongly associated with small primary tumors that present with early distant metastases. The biological mechanisms underlying these patterns—particularly processes related to loss of adhesion, migration, and successful implantation at distant sites—remain the subject of ongoing investigation and are likely to refine future risk-stratification models and multimodal treatment strategies. Future prospective studies should aim to validate these findings in larger, more homogeneous cohorts, integrating molecular and immunological biomarkers, standardized therapeutic algorithms, and real-world clinical endpoints.

## Figures and Tables

**Table 1:** Patients’ characteristics.

	cT1-T2cN1-3cM1 (*n* = 280)		cT3-T4cN0cM0 (*n* = 95)		*p*-Value
	Mean	SD	Mean	SD	
age in years	64.0	10.9	68.4	9.7	0.001
	*n*	%	*n*	%	*p*-value
**sex**					
male	162	57.9%	60	63.2%	
female	118	42.1%	35	36.8%	0.43
smoking status					
never	46	16.4%	4	4.2%	
former	65	23.2%	29	30.5%	
current	127	45.4%	48	50.5%	
unknown	42	15.0%	14	14.7%	0.02
ECOG					
0	151	53.9%	52	54.7%	1.00
1	101	36.1%	34	35.8%	1.00
2	19	6.8%	8	8.4%	0.76
3	3	1.1%	1	1.1%	1.00
unknown	6	2.1%	0	0.0%	0.34

**Table 2:** Tumor characteristics.

	cT1-T2cN1-3cM1 (*n* = 280)		cT3-T4cN0cM0 (*n* = 95)		*p*-Value
	Mean	SD	Mean	SD	
CRP	4.6	9	7.9	23.8	0.2
NRL	3.8	4.9	3.5	4.9	0.54
LDH	291.9	296	170.9	126	<0.0001
	*n*	%	*n*	%	
histological type					
adenocarcinoma	197	70.4%	33	34.7%	<0.0001
squamous-cell carcinoma	25	8.9%	45	47.4%	<0.0001
SCLC	29	10.4%	2	2.1%	0.01
undifferentiated	15	5.4%	7	7.4%	0.46
Net/NEC	7	2.5%	2	2.1%	1.00
LCLC	3	1.1%	3	3.2%	0.17
sarcomatoid	0	0.0%	1	1.1%	0.25
pleomorph carcinoma	1	0.4%	1	1.1%	0.44
adenocystic carcinoma	1	0.4%	1	1.1%	0.44
adenosquamous	2	0.7%	0	0.0%	1.00
TTF1					
positive	162	57.8%	19	20.0%	
negative	69	24.6%	32	33.7%	
unknown	49	17.6%	44	46.3%	<0.00001
Grading					
1	6	2.1%	1	1.0%	
2	36	12.8%	22	23.1%	
3	144	51.4%	33	34.7%	
unknown	94	33.7%	39	41.2%	0.01
LDH					
<100	40	14.3%	27	28.4%	
100 to 249	106	37.9%	51	53.7%	
≥250	134	47.8%	17	17.9%	<0.0001
mutations					
EGFR	37	13.2%	2	2.1%	<0.0001
ALK	15	5.4%	0	0.0%	<0.0001
TP53	9	3.2%	7	7.4%	0.14

**Table 3:** Treatment Appropriateness.

	cT1-T2cN1-3cM1 (*n* = 280)		cT3-T4cN0cM0 (*n* = 95)		*p*-Value
	*n*	%	*n*	%	
therapy appropriate					
yes	177	63.2%	62	65.3%	
no	102	36.4%	33	34.7%	0.84
missing	1	0.4%		0.0%	
NGS therapy appropriate					
yes	171	61.1%			
no	106	37.9%			
missing	3	1.1%			
definitive/palliative local treatment					
definitive local			94	98.9%	
palliative			1	1.1%	

**Table 4:** Logistic regression of survival.

Logistic Regression—T2/T1 vs. T3/T4							
	OR	CI Lower	CI Upper	Coef	SE	z-Value	*p*-Value
age in years	0.97	0.94	1.00	−0.03	0.02	−2.01	0.04
male vs. female	1.40	0.73	2.69	0.33	0.33	1.01	0.31
former vs. never smoker	0.19	0.03	0.74	−1.66	0.76	−2.17	0.03
current vs. never smoker	0.20	0.04	0.72	−1.62	0.74	−2.20	0.03
unknown vs. never smoker	0.29	0.05	1.24	−1.24	0.80	−1.55	0.12
ECOG 1 vs. ECOG 0	1.65	0.84	3.33	0.50	0.35	1.44	0.15
ECOG 2 vs. ECOG 0	2.51	0.79	8.49	0.92	0.60	1.52	0.13
other histology vs. Adenocarcinoma	0.44	0.17	1.19	−0.82	0.50	−1.65	0.10
SCC vs. Adenocarcinoma	0.11	0.04	0.25	−2.24	0.45	−5.02	0.00
SCLC vs. Adenocarcinoma	3.67	0.82	2.73	1.30	0.86	1.51	0.13
TTF1 positive vs. negative	1.61	0.68	3.79	0.48	0.44	1.10	0.27
TTF1 unknown vs. negative	0.37	0.17	0.81	−0.98	0.40	−2.46	0.01
grading 2 vs. 1	0.37	0.01	3.80	−1.00	1.46	−0.69	0.49
grading 3 vs. 1	0.72	0.01	7.32	−0.32	1.45	−0.22	0.82
grading missing vs. 1	0.76	0.01	7.66	−0.27	1.45	−0.19	0.85
CRP	0.97	0.95	0.99	−0.03	0.01	−2.30	0.02
NRL	1.01	0.94	1.08	0.01	0.04	0.22	0.82
LDH ≥ 250 vs. LDH < 100	8.53	3.51	2.19	2.14	0.47	4.61	0.00
LDH 100 to 250 vs. LDH < 100	2.84	1.24	6.64	1.05	0.43	2.46	0.01

**Table 5:** Cox regression of Progression-free survival.

	HR	CI Lower	CI Upper	Coef	SE	z-Value	*p*-Value
T1/T2 vs. T3/T4	6.58	3.96	10.94	1.88	0.26	7.26	<0.0001
age in years	0.99	0.97	1.00	−0.01	0.01	−1.36	0.17
male vs. female	1.24	0.91	1.68	0.21	0.16	1.38	0.17
former vs. never smoker	1.67	1.04	2.69	0.51	0.24	2.12	0.03
current vs. never smoker	1.61	1.02	2.55	0.48	0.23	2.05	0.04
unknown vs. never smoker	1.16	0.66	2.04	0.15	0.29	0.51	0.61
ECOG 1 vs. ECOG 0	1.04	0.76	1.42	0.04	0.16	0.24	0.81
ECOG 2 vs. ECOG 0	1.29	0.67	2.47	0.25	0.33	0.76	0.45
other histology vs. Adenocarcinoma	1.18	0.70	1.97	0.16	0.26	0.62	0.54
SCC vs. Adenocarcinoma	1.03	0.59	1.81	0.03	0.29	0.10	0.92
SCLC vs. Adenocarcinoma	1.56	0.93	2.64	0.45	0.27	1.67	0.09
TTF1 positive vs. negative	0.81	0.54	1.23	−0.21	0.21	−0.98	0.33
TTF1 unknown vs. negative	1.03	0.65	1.62	0.03	0.23	0.11	0.91
grading 2 vs. 1	1.24	0.39	3.96	0.22	0.59	0.37	0.71
grading 3 vs. 1	1.15	0.38	3.49	0.14	0.57	0.24	0.81
grading missing vs. 1	1.10	0.36	3.38	0.09	0.57	0.17	0.87
CRP	1.01	1.00	1.02	0.01	0.00	2.71	0.01
NRL	1.01	0.98	1.04	0.01	0.01	0.86	0.39
LDH ≥ 250 vs. LDH < 100	0.76	0.51	1.15	−0.27	0.21	−1.29	0.20
LDH 100 to 250 vs. LDH < 100	0.51	0.33	0.79	−0.67	0.22	−3.05	0.002
apropriate therapy vs. Not	0.72	0.51	1.01	−0.33	0.17	−1.91	0.06

**Table 6:** Cox regression of Overall survival.

	HR	CI Lower	CI Upper	Coef	SE	z-Value	*p*-Value
T1/T2 vs. T3/T4	2.22	1.42	3.46	0.80	0.23	3.52	0.0004
age in years	1.01	1.00	1.03	0.01	0.01	1.60	0.11
male vs. female	1.51	1.10	2.07	0.41	0.16	2.57	0.01
former vs. never smoker	2.14	1.22	3.77	0.76	0.29	2.65	0.01
current vs. never smoker	2.25	1.31	3.85	0.81	0.27	2.95	0.003
unknown vs. never smoker	2.21	1.18	4.12	0.79	0.32	2.49	0.01
ECOG 1 vs. ECOG 0	1.17	0.84	1.64	0.16	0.17	0.91	0.36
ECOG 2 vs. ECOG 0	3.90	2.31	6.57	1.36	0.27	5.10	<0.0001
other histology vs. Adenocarcinoma	0.77	0.44	1.32	−0.27	0.28	−0.96	0.34
SCC vs. Adenocarcinoma	0.72	0.41	1.25	−0.34	0.29	−1.17	0.24
SCLC vs. Adenocarcinoma	1.54	0.86	2.76	0.43	0.30	1.45	0.15
TTF1 positive vs. negative	0.77	0.49	1.19	−0.27	0.22	−1.19	0.23
TTF1 unknown vs. negative	0.88	0.55	1.41	−0.13	0.24	−0.54	0.59
grading 2 vs. 1	0.84	0.27	2.56	−0.18	0.57	−0.31	0.76
grading 3 vs. 1	0.79	0.27	2.27	−0.24	0.54	−0.45	0.66
grading missing vs. 1	0.60	0.20	1.75	−0.51	0.55	−0.94	0.35
CRP	1.00	0.98	1.01	0.00	0.01	−0.44	0.66
NRL	1.02	0.99	1.05	0.02	0.02	0.99	0.32
LDH ≥ 250 vs. LDH < 100	1.65	1.05	2.60	0.50	0.23	2.17	0.03
LDH 100 to 250 vs. LDH < 100	0.81	0.50	1.31	−0.21	0.25	−0.85	0.39
apropriate therapy vs. Not	0.83	0.59	1.18	−0.18	0.18	−1.04	0.30

## Data Availability

The datasets generated and/or analyzed during the current study are available from the corresponding author on reasonable request.

## Supplementary Materials

The following supporting information can be downloaded at: https://www.mdpi.com/article/10.3390/cancers17233728/s1, Figure S1: Kaplan-Meier curve of survival NSCLC histology; Figure S2: Kaplan-Meier curve of survival SCLC histology; Table S1: Patients’ characteristics according to histology; Table S2: Tumor’s characteristics according to histology; Table S3: Therapy appropriateness based on histology.

## Abbreviations

AJCC	American Joint Committee on Cancer
ALK	Anaplastic Lymphoma Kinase
CUP	Cancer of Unknown Primary
CRP	C-Reactive Protein
ECOG	Eastern Cooperative Oncology Group (Performance Status)
EGFR	Epidermal Growth Factor Receptor
EMT	Epithelial-to-mesenchymal transition
HR	Hazard Ratio
LDH	Lactate Dehydrogenase
LMU	Ludwig Maximilian University
NLR	Neutrophil-to-Lymphocyte Ratio
NSCLC	Non-Small Cell Lung Cancer
OR	Odds Ratio
OS	Overall Survival
P53	Tumor Protein 53
PFS	Progression-Free Survival
PS	Performance Status
SCLC	Small Cell Lung Cancer
Sd	Stable Disease
TTF1	Thyroid Transcription Factor 1
TNM	Tumor, Node, Metastasis
