# Supplementary material for: Distinct Clinical and Biological Features of Diffusely Metastatic Versus Bulky Localized Lung Cancer: Real-World Outcomes from a University Cancer Center in Germany
Source: Cancers (Basel). 2025 Nov 21;17(23):3728. doi: 10.3390/cancers17233728 (PMC12691303; doi:10.3390/cancers17233728)
Supplement: Supplementary file 1 [file cancers-17-03728-s001.zip › cancers-3947740-supplementary.pdf]

Supplementary Materials

Table S1. Patients' characteristics according to histology.

NSCLC					
	T1 or T2 (n = 251)		T3 or T4 (n = 93)		p-value
	mean	sd	mean	sd	
age in years	65.0	11.1	68.6	9.6	0.0004
	n	%	n	%	p-value
sex					
male	146	58.2%	60	64.5%	
female	105	41.8%	33	35.5%	0.35
smoking status					
never	45	17.9%	4	4.3%	
former	57	22.7%	29	31.2%	
current	107	42.6%	46	49.5%	
unknown	42	16.7%	14	15.1%	0.001
ECOG					
0	135	53.8%	50	53.8%	1.00
1	91	36.3%	34	36.6%	1.00
2	17	6.8%	8	8.6%	0.72
3	2	0.8%	1	1.1%	1.00
unknown	6	2.4%	0	0.0%	0.19
SCLC					
	T1 or T2 (n = 29)		T3 or T4 (n = 2)		p-value
	mean	sd	mean	sd	
age in years	64.6	8.6	60.0	12.7	0.001
	n	%	n	%	p-value
sex					
male	16	55.2%	0	0.0%	
female	13	44.8%	2	100.0%	0.23
smoking status					
never	1	3.4%	0	0.0%	
former	8	27.6%	0	0.0%	
current	20	69.0%	2	100.0%	1.00
ECOG					
0	16	55.2%	2	100.0%	0.50
1	10	34.5%	0	0.0%	1.00
2	2	6.9%	0	0.0%	1.00
3	1	3.4%	0	0.0%	1.00

Table S2. Tumor´s characteristics according to histology.

**NSCLC**					
	T1 or T2 (n = 251)		T3 or T4 (n = 93)		p-value
	mean	sd	mean	sd	
CRP	4.6	9	7.9	23.8	0.2
NRL	3.8	4.9	3.5	4.9	0.54

LDH	291.9	296	170.9	126	<0.0001	***
	n	%	n	%		
histological type						
adenocarcinoma	197	78.5%	33	35.5%	<0.0001	***
squamous-cell carcinoma	25	10.0%	45	48.4%	<0.0001	***
undifferentiated	15	6.0%	7	7.5%	0.62	
Net/NEC	7	2.8%	2	2.2%	1.00	
LCLC	3	1.2%	3	3.2%	0.35	
sarcomatoid	0	0.0%	1	1.1%	0.27	
pleomorph carcinoma	1	0.4%	1	1.1%	0.47	
adenocystic carcinoma	1	0.4%	1	1.1%	0.47	
adenosquamous	2	0.8%	0	0.0%	1.00	
TTF1						
positive	153	61.0%	19	20.4%		
negative	54	21.5%	32	34.4%		
unknown	44	17.5%	42	45.2%	<0.00001	***
grading						
1	6	2.4%	1	1.1%		
2	35	13.9%	22	23.7%		
3	119	47.4%	31	33.3%		
unknown	91	36.3%	39	41.9%	0.04	*
LDH						
< 100	35	13.9%	26	28.0%		
100 to 249	97	38.6%	50	53.8%		
>= 250	119	47.4%	17	18.3%	<0.0001	
mutations						
EGFR	37	14.7%	2	2.2%	<0.0001	***
ALK	15	6.0%	0	0.0%	<0.0001	***
TP53	9	3.6%	7	7.5%	0.15	

SCLC	T1 or T2 (n = 29)		T3 or T4 (n = 2)		p-value	
	mean	sd	mean	sd		
CRP	4.6	9	7.9	23.8	0.2	
NRL	3.8	4.9	3.5	4.9	0.54	
LDH	291.9	296	170.9	126	<0.0001	***
	n	%	n	%		
TTF1						
positive	9	31.0%	0	0.0%		
negative	15	51.7%	0	0.0%		
unknown	5	17.2%	2	100.0%	0.05	*
grading						
1	3	10.3%	0	0.0%		
2	1	3.4%	0	0.0%		
3	25	86.2%	2	100.0%	1.00	
LDH						
< 100	5	17.2%	1	50.0%		
100 to 249	9	31.0%	1	50.0%		
>= 250	15	51.7%	0	0.0%	0.26	
mutations						
EGFR	0	0.0%	0	0.0%	NA	
ALK	0	0.0%	0	0.0%	NA	
TP53	0	0.0%	0	0.0%	NA	

Table S3. Therapy appropriateness based on histology.

**NSCLC**					
	T1 or T2 (n = 251)		T3 or T4 (n = 93)		p-value
	n	%	n	%	
therapy appropriate					
yes	150	59.8%	60	64.5%	
no	100	39.8%	33	35.5%	0.52
missing	1	0.4%		0.0%	
NGS therapy appropriate					
yes	145	57.8%			
no	103	41.0%			
missing	3	1.2%			
definitive/palliative local treatment					
definitive local			92	98.9%	
palliative			1	1.1%	
SCLC					
	T1 or T2 (n = 29)		T3 or T4 (n = 2)		p-value
	n	%	n	%	
therapy appropriate					
yes	27	93.1%	2	100.0%	
no	2	6.9%	0	0.0%	1.00
NGS therapy appropriate					
yes	26	89.7%			
no	3	10.3%			
definitive/palliative local treatment					
definitive local			2	100.0%	
palliative			0	0.0%	

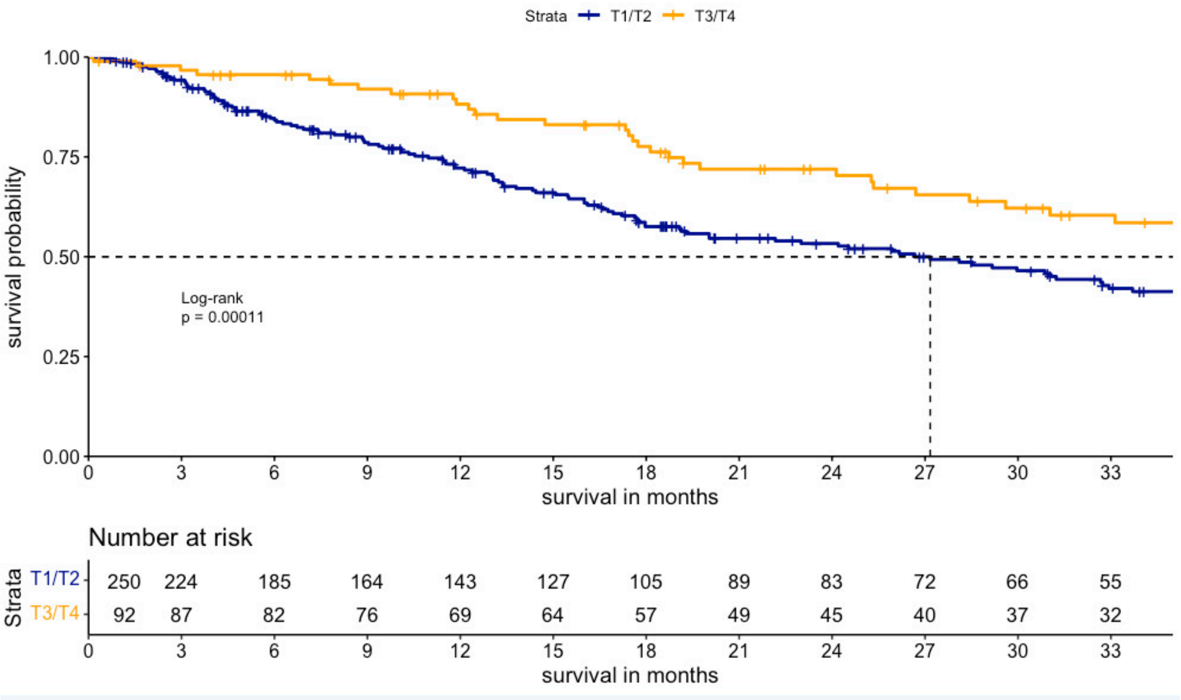

Figure S1. Kaplan-Meier curve of survival NSCLC histology.

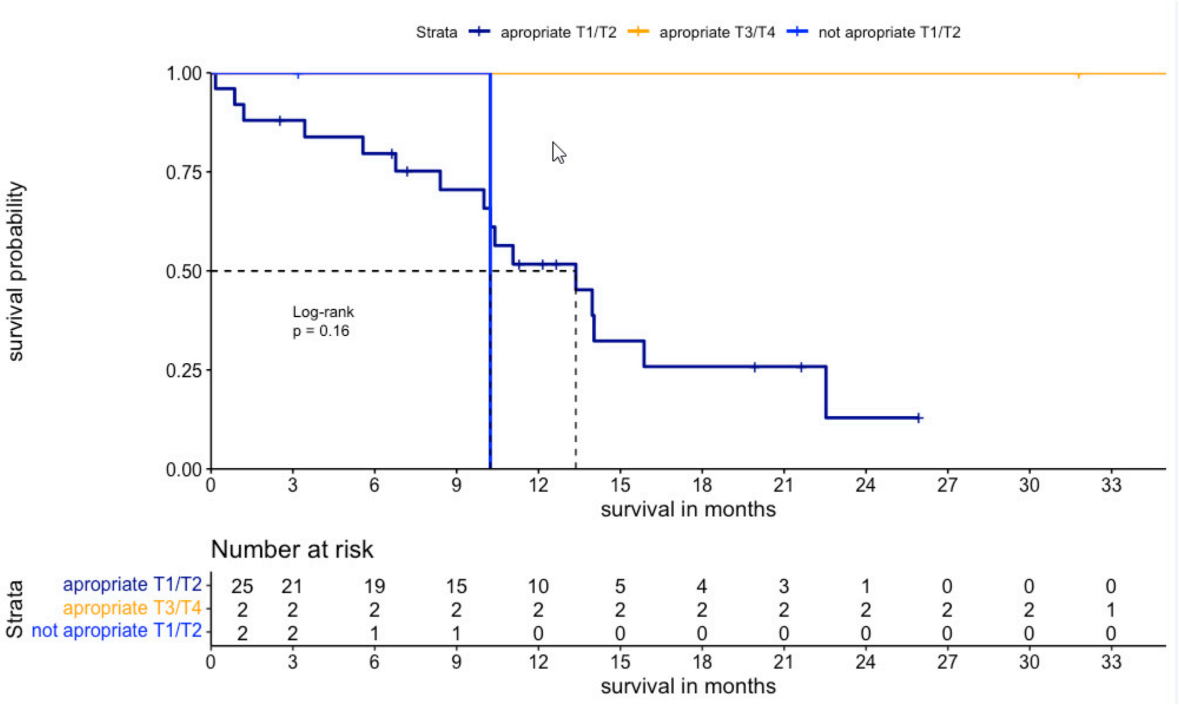

Figure S2. Kaplan-Meier curve of survival SCLC histology.
